# Significant Performance and Stability Improvements of Low-Temperature IGZO TFTs by the Formation of In-F Nanoparticles on an SiO_2_ Buffer Layer

**DOI:** 10.3390/nano10061165

**Published:** 2020-06-15

**Authors:** Ho-young Jeong, Seung-hee Nam, Kwon-shik Park, Soo-young Yoon, Chanju Park, Jin Jang

**Affiliations:** 1 Advanced Display Research Center, Department of Information Display, Kyung Hee University, Dongdaemun-gu, Seoul 130-701, Korea; hyjeong@lgdisplay.com (H.-y.J.); cjpark@tft.khu.ac.kr (C.P.); 2 LG Display R&D Center, 245, Lg-ro, Paju-si, Gyeonggi-do 413-811, Korea; shnam@lgdisplay.com (S.-h.N.); pius@lgdisplay.com (K.-s.P.); syyoon@lgdisplay.com (S.-y.Y.)

**Keywords:** low-temperature coplanar IGZO TFT, bias stability, In-F nanoparticles

## Abstract

We report the performance improvement of low-temperature coplanar indium–gallium–zinc–oxide (IGZO) thin-film transistors (TFTs) with a maximum process temperature of 230 °C. We treated F plasma on the surface of an SiO_2_ buffer layer before depositing the IGZO semiconductor by reactive sputtering. The field-effect mobility increases from 3.8 to 9.0 cm^2^ V^−1^·s^−1^, and the threshold voltage shift (ΔV_th_) under positive-bias temperature stress decreases from 3.2 to 0.2 V by F-plasma exposure. High-resolution transmission electron microscopy and atom probe tomography analysis reveal that indium fluoride (In-F) nanoparticles are formed at the IGZO/buffer layer interface. This increases the density of the IGZO and improves the TFT performance as well as its bias stability. The results can be applied to the manufacturing of low-temperature coplanar oxide TFTs for oxide electronics, including information displays.

## 1. Introduction

Indium–gallium–zinc–oxide (IGZO) thin-film transistors (TFTs) are widely used as a TFT backplane for active-matrix liquid-crystal displays (AMLCDs) and active-matrix organic light-emitting diode (AMOLED) displays due to their higher mobility and smaller subthreshold swing compared to amorphous silicon (a-Si) TFTs [1,2,3,4]. To realize the future of displays with flexible and transparent functions, transparent polyimide (PI) and poly(ethersulfone) (PES) could be used, and a low-temperature process (~230 °C) is required. The advantage of oxide TFTs is that they require a low-temperature process, but a relatively high-temperature process at about 350 °C is being used for the mass production of displays. This is mainly due to the improvement of the mobility and bias stability of oxide TFTs [5,6]. It is noted that the quality of SiO_2_ depends on its substrate process temperature; the density and leakage through SiO_2_ and electrical breakdown voltage could be improved by increasing the deposition temperature of SiO_2_. The buffer SiO_2_ of coplanar structures and the gate dielectric SiO_2_ of back-channel-etch (BCE) structures are usually deposited at 300 to 400 °C by plasma-enhanced chemical vapor deposition (PECVD) for display applications [7,8]. The coplanar structure of IGZO TFTs has the advantage of negligible overlap capacitance between the gate and source/drain electrodes, and thus, the RC (resistance–capacitance product) delay can be remarkably reduced for display applications [5,6,7] [9,10,11]. In addition, the coplanar oxide TFT is a strong candidate for low-temperature devices because it could not use high-temperature SiO_2_ as a gate insulator (GI) because of the damage this would cause to IGZO during GI deposition [12]. In order words, coplanar oxide TFTs with only a high-temperature buffer layer have good performance to be used in AMOLED televisions [13,14], and the coplanar oxide TFTs with a low-temperature buffer layer could be widely used as low-temperature TFTs, if the TFTs improve their performance. Note that low-temperature coplanar IGZO TFTs suffer from lower mobility and higher threshold voltage shift (ΔV_th_) under positive-bias temperature stress (PBTS), and improving PBTS stability is important for the development of stable IGZO TFTs for AMOLED displays, given that driving TFTs in the OLED pixel are positively biased, and even a small change in the V_th_ can deteriorate image quality [15,16].

In this work, we studied the effect of F-plasma treatment on low-temperature SiO_2_ to improve the performance and bias stability, PBTS, of the low-temperature coplanar IGZO TFT. The physical damage by ion bombardment on the SiO_2_ buffer layer during the sputter deposition of IGZO might be one of the main reasons for the generation of trap sites in low-temperature SiO_2_ [17]. To reduce the trap sites generated during the sputtering process, we performed F-plasma treatment on the SiO_2_ buffer because F is known to passivate traps in SiO_2_ as well as in IGZO [18,19,20,21,22]. Methods such as dynamic secondary-ion mass spectroscopy (D-SIMS), high-resolution transmission electron microscopy (HR-TEM) and APT (atom probe tomography) were used to investigate the relationships between the process temperature of the buffer layer, the performance of the TFTs and the effect of plasma treatment on the buffer layer.

## 2. Materials and Methods

**Figure 1:**
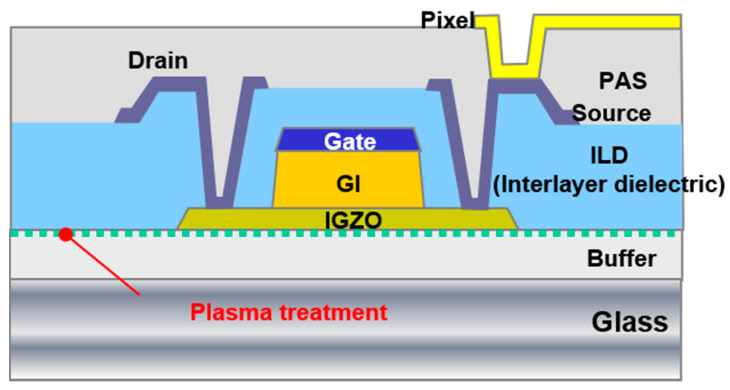
Cross-sectional view of the coplanar indium–gallium–zinc–oxide (IGZO) thin-film transistor (TFT) studied in this work.

The cross-sectional view of the coplanar IGZO TFT studied in this work is shown in Figure 1. First, a 200-nm-thick SiO_2_ was deposited as buffer layer at 230 or 350 °C by PECVD (Applied Materials, Santa Clara, CA, USA). The F-plasma treatment (500 W, 100 mTorr, 60 s) was carried out in a reactive ion etching (RIE) chamber before depositing the IGZO. Device I is the control sample with a high-temperature buffer layer processed at 350 °C. Device II is another control sample with a low-temperature SiO_2_ buffer layer processed at 230 °C. Device III is for checking the effect of F-plasma treatment on the buffer layer processed at 230 °C. A 30-nm IGZO (In: Ga: Zn = 1: 1: 1 at%) active layer (JX NIPPON MINING & METALS KOREA Co., Ltd, Pyeongtaek, Korea) was deposited by direct current sputtering at room temperature. Then, a 200-nm-thick SiO_2_ layer was deposited by PECVD at 230 °C as the gate insulator (GI). The subsequent thermal annealing was performed at 230 °C for 1 h in air and followed by the sequential deposition of a 300-nm-thick Mo layer as the gate metal. The Mo and GI layers were patterned continuously by photolithography. He-plasma treatment was applied to make n^+^ ohmic contacts with source/drain electrodes as previously reported [21,23]. A 400-nm-thick SiO_2_ was deposited by PECVD at 230 °C to form the interlayer dielectric (ILD). Contact holes were formed by dry etching, after which a 300-nm-thick Mo layer was sputtered and then patterned by wet etching to form the source/drain electrode. A 200-nm-thick SiO_2_ layer was deposited at 230 °C as a passivation layer by PECVD and then patterned. Finally, an indium tin oxide (ITO) was deposited and wet etched to form the pixel electrodes.

## 3. Results and Discussions

**Figure 2:**
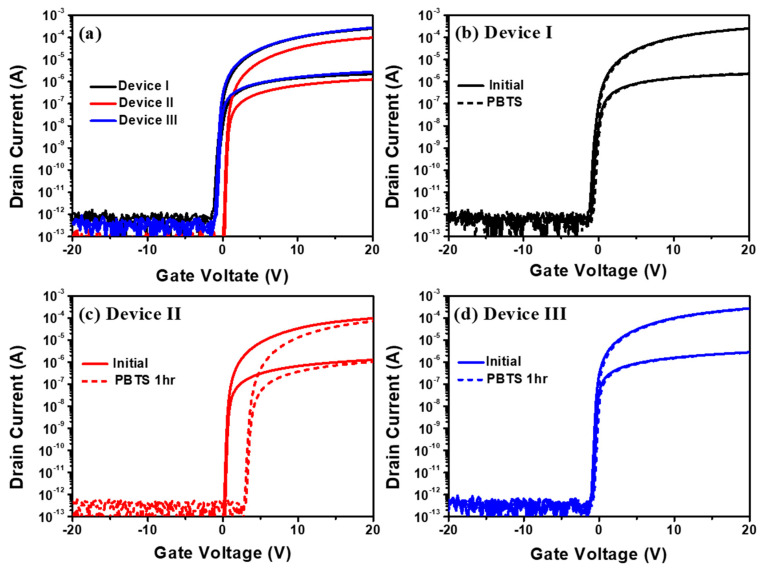
(**a**) Transfer curves of the TFTs I, II and III (W/L = 100/10 μm) before bias stress. The transfer curves were measured before and after 1 h of PBTS (dash line) at V_DS_ = 0.1 and 10 V for (**b**) device I, (**c**) device II and (**d**) device III, respectively.

Figure 2a shows the transfer characteristics of the devices before stress. Figure 2b–d shows the transfer characteristics of devices I, II and III before and after 1 h of PBTS, applying a constant gate voltage (V_GS_) of +30 V at 60 °C for 1 h in a dark box. The channel width and length of the devices are 100 and 10 μm, respectively. Table 1 lists the device parameters, such as threshold voltage (V_th_), field-effect mobility (µ_FE_), subthreshold swing (SS) and V_th_ shift (ΔV_th_) under PBTS for the three devices. V_th_ is the V_GS_ giving the drain current (I_D_) = W/L × 10 nA at the drain voltage (V_DS_) = 10 V. µ_FE_ is obtained at V_GS_–V_th_ = 10 V and V_DS_ = 10 V. The SS is taken from the minimum value of ΔV_GS_/Δlog(I_DS_) with V_DS_ = 10 V. Devices I and II are fabricated with the same process conditions, except for the process temperature of the buffer layer. However, the mobility increases by 2.4 times and the V_th_ shift decreases from 3 to 0.2 V by using a high-temperature buffer layer. For device III, the initial V_th_, SS, µ_FE_ and ΔV_th_ are found to be −0.2 V, 139 mV/dec, 9 cm^2^ V^−1^·s^−1^ and 0.2 V, respectively. The results show that the performance of device III is similar to that of device I, and that plasma treatment plays a critical role to improve performance.

**Figure 3:**
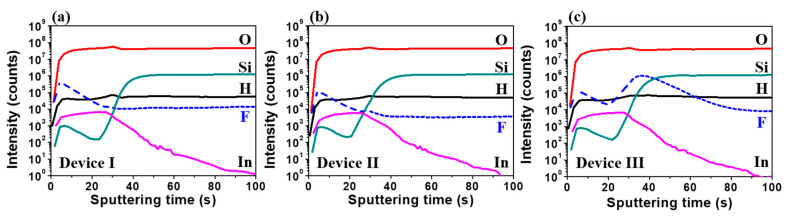
The dynamic secondary-ion mass spectroscopy (D-SIMS) profiles of H, O, F, Si and In for devices (**a**) I, (**b**) II and (**c**) III, respectively.

Figure 3a–c shows the D-SIMS profiles of H, O, F, Si and In for devices I, II and III, respectively. The significant increase of F intensity was found for device III. This result shows that F is incorporated into the IGZO/SiO_2_ interlayer. The presence of F in the top region of the active layer of the TFT appears to be due to the contamination from the chamber wall because NF_3_ plasma is used to etch the deposited layers to clean the PECVD chambers.

**Figure 4:**
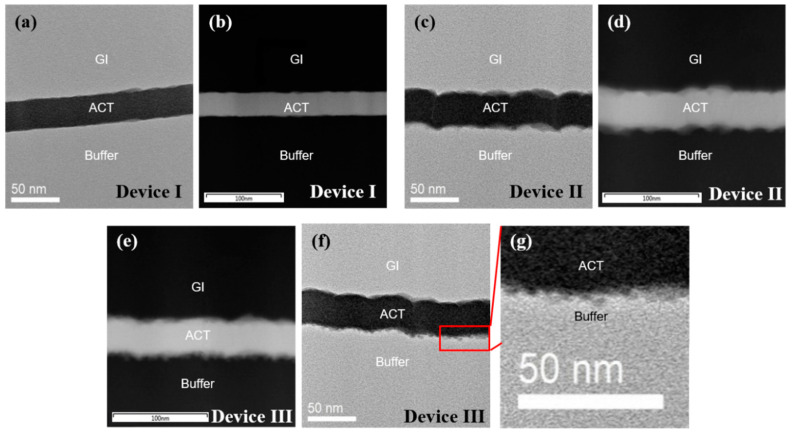
High-resolution TEM (HR-TEM) images of the gate insulator (GI) (SiO_2_)/active (IGZO)/buffer (SiO_2_) interfaces for devices (**a**) I, (**c**) II and (**f**) III, respectively. Annular dark-field (ADF) images in a scanning TEM (STEM) for devices (**b**) I, (**d**) II and (**e**) III, respectively. (**g**) Enlarged interface of device III.

Figure 4 shows the cross-sectional HR-TEM images and annular dark-field (ADF) images in a scanning TEM (STEM) of the GI (SiO_2_)/IGZO/buffer (SiO_2_) interfaces. Figure 4a shows that device I has smooth surface roughness at the top and bottom regions of the active layer. This relates to a buffer layer fabricated through a high-temperature process. On the other hand, device II has poor roughness (~8 nm RMS roughness) at the bottom of the active layer, caused by the low-temperature buffer layer. Therefore, the top IGZO surface has poor surface morphology (Figure 4c). The results show the roughness of the GI/IGZO is affected by the deposition temperature of the buffer layer. Device III also has poor roughness (Figure 4f), but nanoparticles are found at the IGZO/Buffer (SiO_2_) interface. To identify nanoparticles, the annular dark-field (ADF) images in a scanning TEM (STEM) of the devices were analyzed and the interface of device III was enlarged. Figure 4b,d,e shows ADF images for each device. ADF imaging is a method of mapping samples in a STEM. These images are formed by collecting scattered electrons with an annular dark-field detector [24]. Note that the sizes of scale bar in Figure 4d,e are larger than in Figure 4b for the identification of the nanoparticles. The nanoparticles, of around 1 nm in diameter, are identified by Figure 4e and the enlarged view of Figure 4g. The SiO_2_ roughness affects the performance of the devices. In addition, the nanoparticles produced by plasma treatment seem to improve the mobility and V_th_ shift by PBTS.

**Figure 5:**
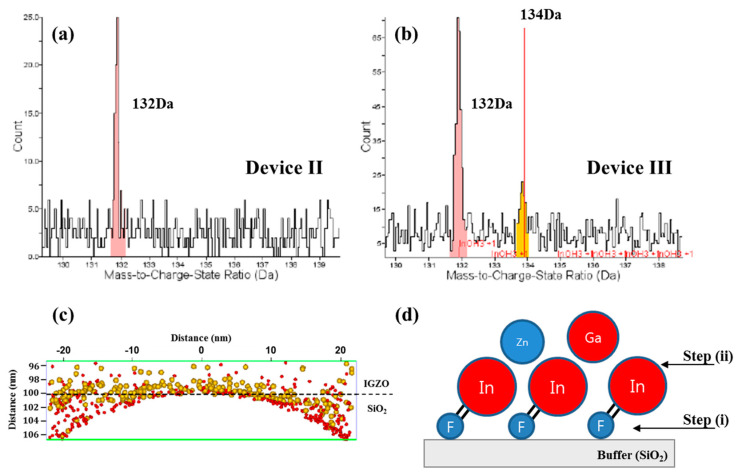
Mass spectra at the IGZO/buffer (SiO_2_) interface for (**a**) device II and (**b**) device III. (**c**) Atomic map for device III (132 Da: red, 134 Da: yellow) analyzed with APT (atom probe tomography). (**d**) InF nanoparticle generation model. The InF formation can be seen in (**d**); step (i) is F bonding on SiO_2_ and step (ii) is the formation of InF nanoparticles during the IGZO sputtering process.

We measured the mass spectra and atomic map by atom probe tomography, APT (CAMECA, LEAP5000). APT supports the material analysis, offering extensive capabilities for both 3D imaging and chemical composition measurements at the atomic scale (around 0.1–0.3 nm resolution in depth and 0.3–0.5 nm laterally) [25]. The IGZO (100 nm)/SiO_2_ (200 nm) layers, deposited with the same process conditions used for fabricating devices II and III, were used for APT measurement. Note that the 100-nm IGZO layer was used to prevent contamination during APT measurement. Figure 5a,b shows the mass spectra for the IGZO/buffer (SiO_2_) interfaces of devices II and III. A 132 Da (Dalton) peak is found for the samples, which may be related with Si_3_O_3_ because of the atomic mass (Si: 28 Da, O: 16 Da). The peak of 134 Da is observed, which is estimated to be InF (In: 115 Da, F: 19 Da). As shown in the atomic map (Figure 5c), the InF nanoparticles of about 1 nm in diameter are formed on the top of Si_3_O_3_. Figure 5d shows the generation model of InF nanoparticles with the F atoms introduced by plasma treatment on the buffer layer. The InF nanoparticles could be generated during the IGZO sputtering process because In has the highest electron affinity and the largest reactivity with F. For example, the electron affinity of In is 37.043 kJ/mol, Ga is 29.061 kJ/mol and Zn is −58 kJ/mol. It is reported that F-plasma treatment on IGZO improves the TFT performance by passivating electron traps in IGZO. We found in this work that F-plasma treatment on the buffer layer formed InF nanoparticles at the IGZO/SiO_2_ interfaces. The nanoparticles increase the density of the active layer in spite of having poor roughness. The increased density of the IGZO can improve the mobility and V_th_ shift by PBTS (Figure 2). As a result, device III has excellent TFT performance even at the low-temperature buffer-layer deposition.

## 4. Conclusions

We report the significant improvement of a low-temperature coplanar IGZO device by plasma exposure on an SiO_2_ buffer layer. The low-temperature IGZO TFT (device III) has similar performance and V_th_ shift for PBTS to those of IGZO TFT with the buffer layer fabricated at 350 °C, by introducing F plasma on the buffer layer (230 °C). By TEM and APT analyses, it is found that In-F nanoparticles of about 1 nm in diameter are formed at the IGZO/buffer interface. Therefore, F-plasma treatment on an SiO_2_ buffer layer can be a suitable method to make low-temperature coplanar IGZO TFTs.

## Figures and Tables

**Table 1:** Summary of the device parameters and bias stability for the three TFTs. The threshold voltage shift (ΔV_th_) was measured after 1 h PBTS.

	V_th_ (V)	µ_FE_ (cm^2^/V·s)	SS (mV/dec)	ΔV_th_ (V)
Device I	0.0	9.2	181	0.2
Device II	1.1	3.8	109	3.2
Device III	−0.2	9.0	139	0.2
